# Effect of hydrogel-spacer implantation and uneven positioning on rectal dose in intensity-modulated radiotherapy for prostate cancer

**DOI:** 10.7150/jca.119694

**Published:** 2025-10-01

**Authors:** Masafumi Uno, Yukihisa Tamaki, Kasumi Nonomura, Unta Yamamori, Hiroshi Burioka, Natsuko Nagano, Atsushi Ue, Yoko Sonoyama, Rika Yoshida, Kazuhiro Kitajima, Koichiro Wada

**Affiliations:** 1 Department of Radiation Oncology, Shimane University Faculty of Medicine, Shimane, Japan.; 2 Department of Radiotherapy, Shimane Prefectural Central Hospital, Shimane, Japan.; 3 Department of Radiology, Shimane University Faculty of Medicine, Shimane, Japan.; 4 Department of Radiology, Hyogo Medical University, Hyogo, Japan.; 5 Department of Urology, Shimane University Faculty of Medicine, Shimane, Japan.

## Abstract

**Objectives:** Implanting a hydrogel spacer in radiation therapy for prostate cancer is effective in reducing rectal dose. Hydrogel spacer can be unevenly distributed. we often encountered in which the hydrogel spacer was not in place at the prostatic apex. This study had two objectives. The first was to analyze whether the rectal dose could be reduced in patients who underwent hydrogel-spacer implantation at our hospital. The second was to analyze whether the rectal dose could be reduced in cases where the hydrogel spacer was unevenly distributed and not in place at the prostatic apex, as compared with cases without hydrogel-spacer implantation.

**Methods:** We retrospectively reviewed the records of patients who underwent intensity-modulated radiation therapy for prostate cancer at our hospital between March 2020 and June 2022. Initially, the rectal dose parameters were compared between patients who underwent hydrogel-spacer implantation and those who did not. Additionally, the same parameters were compared between patients who did not undergo hydrogel-spacer implantation and those who did, but in whom the spacer was not in place at the apex.

**Results:** 45 patients did not undergo hydrogel-spacer implantation and 36 patients did. A comparison of rectal dose parameters between patients with and without hydrogel-spacer implantation showed a reduction in all parameters in those with implantation. The 36 patients with hydrogel-spacer implantation included 16 patients in whom the hydrogel spacer was not in place at the apex. A comparison of rectal dose parameters between the 45 patients without hydrogel-spacer implantation and the 16 patients with the hydrogel spacer not in place at the apex showed a reduction in all parameters in the latter group.

**Conclusion:** Hydrogel-spacer implantation was effective in reducing the rectal dose. The rectal dose could be reduced even in cases with uneven distribution of the spacer, as compared with cases without spacer implantation.

## Introduction

Prostate cancer is the most common type of cancer in men in Japan 1, a typical treatment for which is external beam radiotherapy. In external beam radiotherapy for prostate cancer, increased radiation dose has been associated with improved biochemical control 2,3. Intensity-modulated radiation therapy (IMRT) is presently the mainstay of radiation therapy for prostate cancer. Compared with conventional three-dimensional conformal radiation therapy (3D-CRT), IMRT can deliver a higher total dose by increasing the dose concentration. In Japan, IMRT combined with long-term androgen therapy has demonstrated good results in terms of 10-year biochemical control 4. In patients with pretreatment prostate-specific antigen >10 ng/mL or high-risk disease, increasing the radiation dose has been shown to improve not only biochemical control but also the overall survival rate 5. Because the prostate is adjacent to organs at risk such as the rectum and bladder, adverse events (AEs) in these adjacent organs, especially in the rectum, are often a problem with radiotherapy. Previous research reported that high-dose treatment with conventional 3D-CRT resulted in late rectal AEs of Grade 2 or higher (according to the Radiation Therapy Oncology Group late radiation morbidity scoring scheme) in approximately 20% of cases 3,6. The introduction of IMRT has increased dose concentration and reduced AEs. However, even with the use of IMRT, the incidence of Grade ≥2 rectal AEs remains around 5%-12% 4,7.

The incidence of rectal AEs is correlated with the irradiated volume of the rectal wall 8-10. However, in prostate cancer radiotherapy, an irradiation margin of several millimeters needs to be established around the prostate. Therefore, a part of the anterior rectal wall is inevitably included in the irradiated range. To reduce rectal AEs, it is important to reduce the irradiated rectal wall volume. One approach to this is to reduce the rectal dose by increasing the physical distance between the prostate and rectum. Various materials have been inserted as part of efforts aimed at reducing the rectal dose 11,12. One such material currently used in Japan is SpaceOAR (Boston Scientific, Marlborough, Massachusetts). SpaceOAR is a polyethylene-glycol hydrogel spacer that is injected between the prostate and the rectum. Implanting a hydrogel spacer between the prostate and rectum has been shown to be effective in reducing rectal AEs 5,6. Shimane University Hospital began performing hydrogel-spacer implantation in January 2020.

In this study, we aimed to answer two questions. The first was whether the rectal dose could be reduced by implanting a hydrogel spacer. Although there have already been several reports discussing the effectiveness of hydrogel spacers 13,14, we also investigated whether hydrogel-spacer implantation would lead to a reduction in the rectal dose at our hospital.

**Figure 1:**
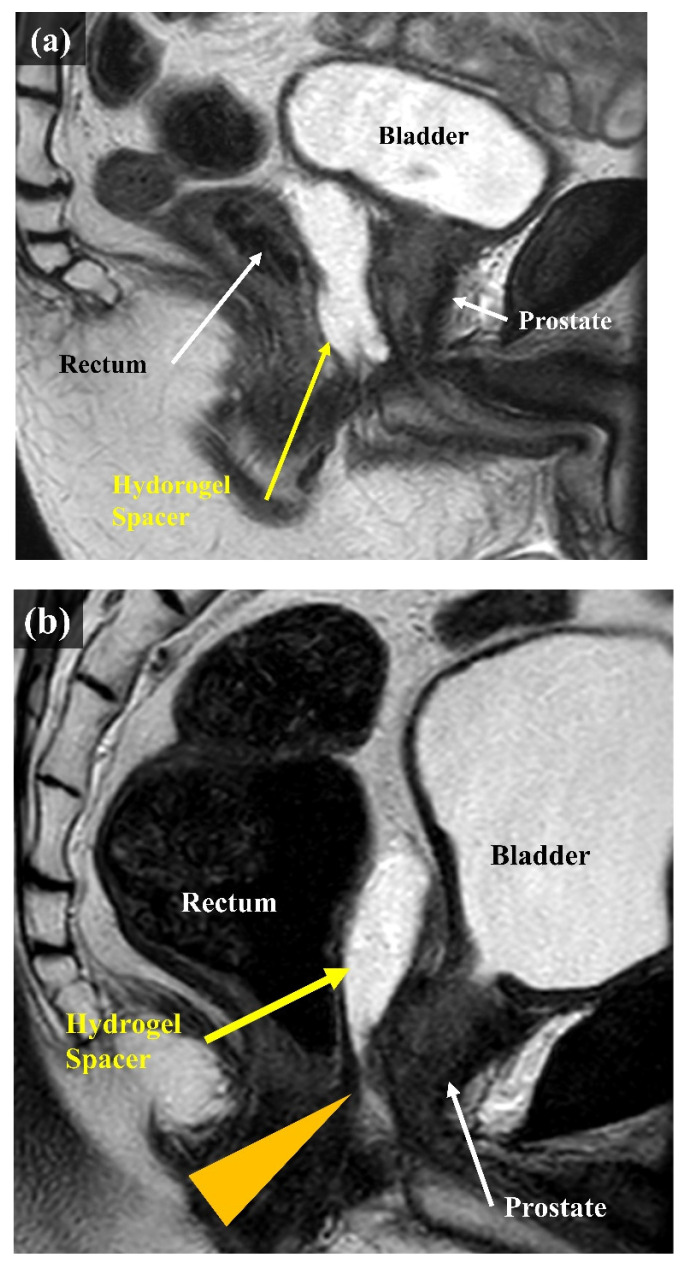
MRI scans (sagittal). (a) Case in which the hydrogel spacer is uniformly distributed from the base to the apex on the dorsal side of the prostate (Apex group). (b) Case in which the spacer is unevenly distributed towards the cranial side of the prostate The arrowhead indicates that the hydrogel spacer is not in place at the apex (Non-Apex group).

The second question was whether the rectal dose could be reduced even if the hydrogel spacer was unevenly distributed in the craniocaudal direction. As we gained more experience with hydrogel-spacer implantation, we encountered multiple cases where the spacer was unevenly distributed. It tends to be unevenly distributed in the craniocaudal direction, with multiple cases encountered in which the hydrogel spacer was not in place at the level of prostatic apex, despite a sufficient spacer thickness being available at the level of the center of the prostate (Figure 1). Reports on proton beam therapy for prostate cancer have shown the effectiveness of hydrogel-spacer implantation in the prostatic apex 15. Meanwhile, no report has described the uneven distribution of the hydrogel spacer at the apex during X-ray-based IMRT. In this study, we examined whether the rectal dose could be reduced in cases where the hydrogel spacer was unevenly distributed and not implanted at the level of prostatic apex, as compared with cases with no hydrogel-spacer implantation.

## Methods and Materials

### Patients

We reviewed the records of patients who underwent radical IMRT for prostate cancer at Shimane University Hospital between March 2020 and June 2022. Patients who underwent radiation therapy after prostate cancer surgery and those who received prophylactic nodal radiation were excluded. We retrospectively analyzed the information collected from the medical records and the radiation treatment planning system. All patients were informed in detail about radiation therapy and hydrogel-spacer implantation, and their written consent was obtained. This study was approved by the Medical Research Ethics Committee, Shimane University Faculty of Medicine (Research Control Number: 20220823-1).

### Hydrogel spacer

The implantation of the hydrogel spacer was carried out in accordance with the “Guideline for Proper Use of SpaceOAR System in Radiation Therapy for Prostate Cancer” published by the Japanese Society for Radiation Oncology 16. At our hospital, eligibility was determined based on this guideline. Patients with extracapsular extension on the dorsal side of the prostate, seminal vesicle invasion, or a bleeding tendency were excluded. In addition, detailed medical histories were reviewed, including history of pelvic surgery and comorbidities such as diabetes, and the final eligibility was determined at a conference involving multiple radiation oncologists. For patients deemed eligible, a detailed explanation of the spacer was provided, and the patient's willingness to undergo the procedure was confirmed before implantation was ultimately performed. The procedure was as follows. Under transrectal ultrasound guidance, a needle was advanced into the perirectal fatty tissue between Denonvillier's fascia and the anterior rectal wall. The needle tip was set at the level of the center of the prostate, in accordance with the aforementioned guidelines. Before injecting the hydrogel spacer, saline solution was injected into the perirectal space for hydro-dissection to confirm that the needle tip was in the appropriate position. At Shimane University Hospital, a urologist places a hydrogel spacer with radiation oncologists. Hydrogel-spacer implantation was performed under local anesthesia in all patients.

### Radiation therapy

At Shimane University Hospital, two treatment-planning computed tomography (CT) scans are taken for radiotherapy treatment planning. The first CT scan was taken 2 to 3 weeks after hydrogel-spacer implantation. CT scans were taken approximately 1-2 hours after the patient's last urination, with the bladder in a filled state. In patients with an implanted hydrogel spacer, magnetic resonance imaging (MRI) was also performed on the same day as the first CT to check the distribution of the spacer. The second CT was taken the week after the first CT. Based on these two CT scans, the radiation oncologist comprehensively assessed the extent of rectal and bladder expansion and their relative positions, and developed a radiotherapy treatment plan.

At Shimane University Hospital, the clinical target volume (CTV) is defined as the prostatic parenchyma plus approximately one-third of the seminal vesicles. This definition of CTV was applied regardless of the risk classification of prostate cancer specified by the National Comprehensive Cancer Network. The exception to this was cases with confirmed seminal vesicle invasion, in which entire seminal vesicles were included in the CTV. The planning target volume (PTV) margin was set at 5 mm dorsally and 8 mm in all other directions. For the evaluation of rectal dose, the rectal wall was contoured. The craniocaudal extent was defined from 1.5 cm cranial to the upper edge of the CTV to the anal verge, and the inner 4 mm of this contour was defined as the rectal wall.

The irradiation method used was fixed-field IMRT, with or without a hydrogel spacer. In all cases, 6-MV X-rays were used. The standard prescribed dose was 78 Gy delivered in 39 fractions of 2 Gy per day. In 1 patient who was classified as low risk according to the NCCN risk classification and declined invasive procedures, treatment was delivered without spacer implantation, at 76 Gy in 38 fractions of 2 Gy per day. Treatment plans were designed to ensure that at least 85% of the PTV received the prescribed dose. When the small intestine had prolapsed into the pelvic cavity, it was sometimes included in or located adjacent to the uniformly defined PTV target. In such cases, priority was given to the dose constraints of the small intestine. A modified PTV target with a reduced margin near the small intestine was created and used for treatment planning. Such a modified PTV was used for 2 patients. Reduction of the rectal dose was achieved by minimizing overall radiation exposure to the rectum. No additional constraints were applied to the rectal wall adjacent to the prostatic apex, regardless of hydrogel spacer implantation or uneven positioning. Radiotherapy was administered once daily. Prior to each treatment, the bladder filling state was confirmed using cone-beam CT or ultrasonography.

### Evaluation methods

In this study, we aimed to answer two research questions. The first question was whether the rectal dose could be reduced by hydrogel-spacer implantation. As with other reports, we also statistically evaluated whether the hydrogel-spacer implantation performed at Shimane University Hospital would reduce the rectal dose. The second question was whether the rectal dose could be reduced even if the hydrogel spacer was unevenly distributed in the craniocaudal direction. In this study, we investigated how much the rectal dose was reduced in cases where the hydrogel spacer was not in place at the prostatic apex. Analyses were performed using JMP Pro ver. 17.0.0 (SAS Institute Inc., Cary, NC, USA). Statistical tests were two-sided and were considered statistically significant at P<0.05.

### Reduction of rectal dose by hydrogel-spacer implantation

The rectal dose data (D2cc, D1cc, V78Gy, V70Gy, V60Gy, and V40Gy) for patients with and without hydrogel-spacer implantation (hereinafter referred to as the Spacer group and Non-Spacer group, respectively) were extracted from the radiotherapy treatment planning system (RTPS). The RTPS used in this study was the Eclipse Treatment Planning System (Eclipse TPS; Varian Medical Systems, Palo Alto, CA, USA). Eclipse TPS ver. 13.6 was used from March 2020 to March 2022, and ver. 16.1 was used from April 2022 onwards. The mean rectal doses were calculated for the Non-Spacer group and Spacer group, and the difference in rectal dose between the groups was statistically analyzed using the Wilcoxon rank sum test.

In addition, rectal dose data with and without hydrogel-spacer implantation in the same patients were analyzed by the same methods. In this retrospective study, treatment-planning CT scans immediately prior to spacer implantation were not available for comparison. Therefore, diagnostic CT scans obtained at the time of prostate cancer diagnosis or other occasions were used for comparison. These CT scans were used to generate simulated treatment plans. As with the actual treatment, the simulation plans were designed to deliver a total dose of 78 Gy in 39 fractions of 2 Gy, ensuring that at least 85% of the PTV received the prescribed dose. In the Spacer group, several patients had undergone hormonal therapy before radiotherapy, resulting in substantial changes in prostate size. For this analysis, we selected cases from the Spacer group in which the change in prostate size between the actual and simulated treatment plans was within 20%. Among the patients extracted, simulated treatment plans based on the CT scans were compared with their actual treatment results. We defined the simulated treatment plans based on pre-spacer CT scans as Group A, and the actual treatment plans using post-spacer treatment planning CT scans as Group B. Rectal dose data (D2cc, D1cc, V78Gy, V70Gy, V60Gy, V40Gy) were extracted for Groups A and B and statistically analyzed using the Wilcoxon signed-rank test.

### Rectal dose in patients with an unevenly distributed hydrogel spacer

**Figure 2:**
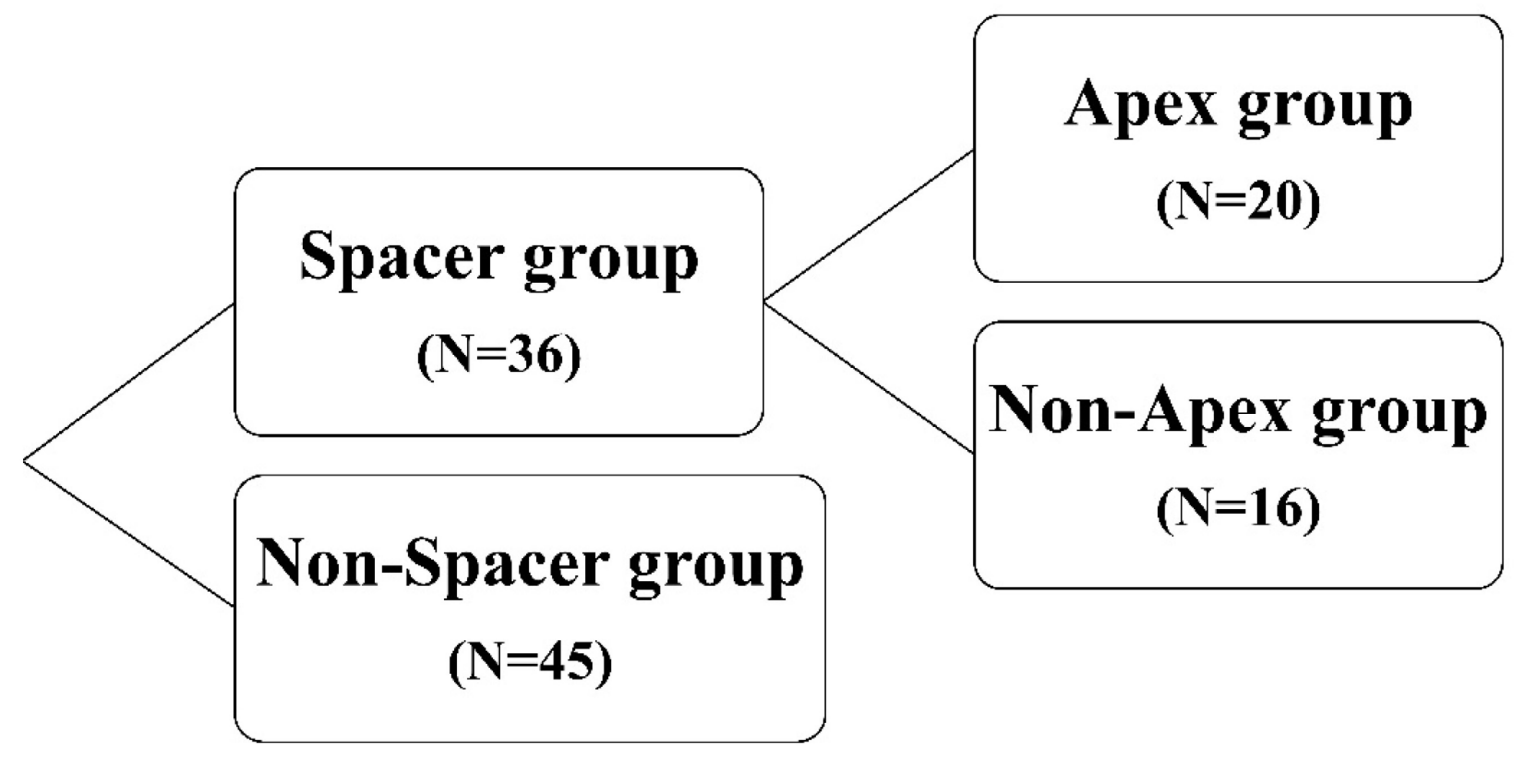
Patients were divided into those who did undergo hydrogel-spacer implantation (Spacer group, n = 36) and those who did not (Non-Spacer group, n = 45). The patients in the spacer group were further divided into those with the hydrogel spacer in place at the prostatic apex (Apex group, n = 20) and those with the hydrogel spacer not in place at the apex (Non-Apex group, n = 16).

**Figure 3:**
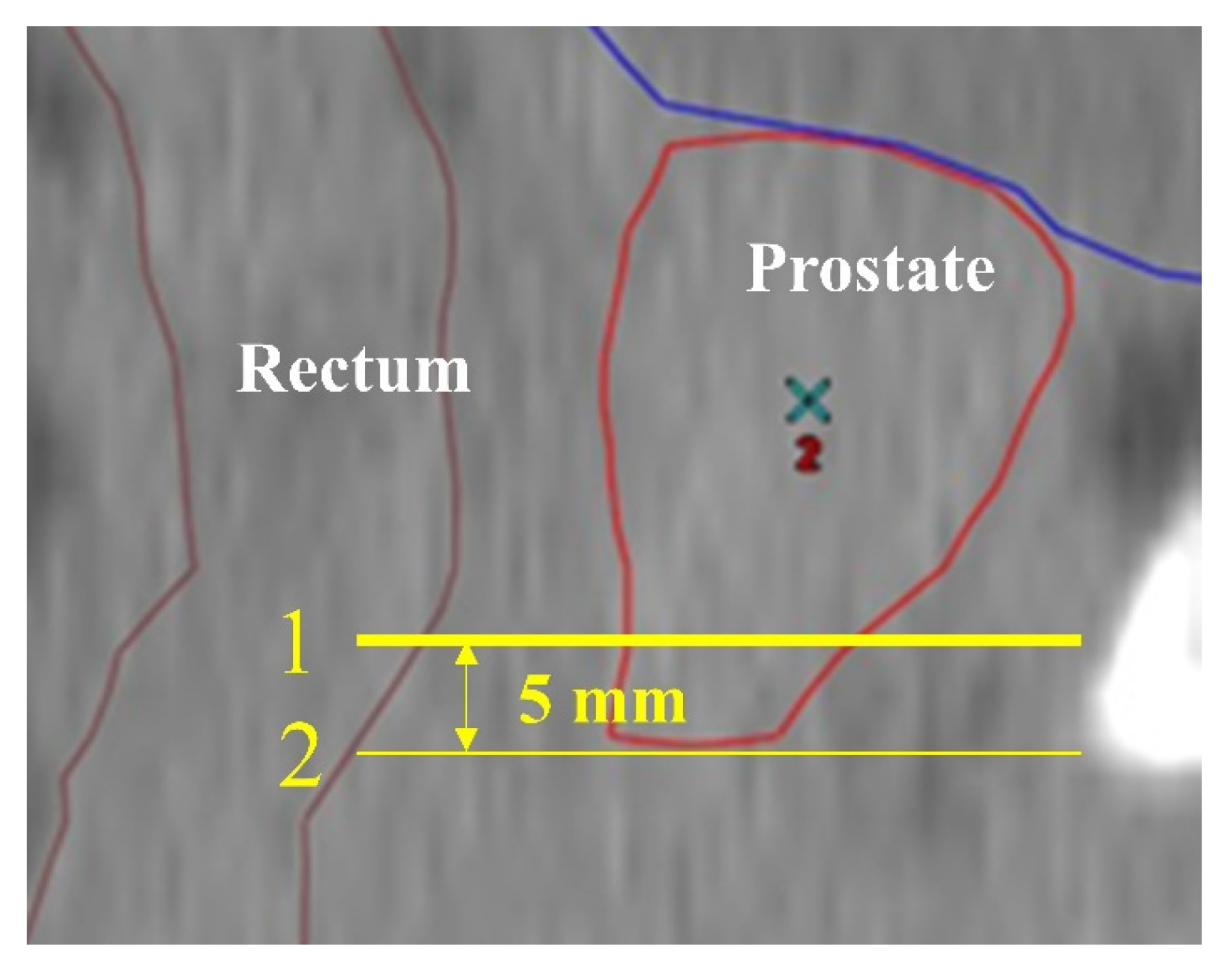
CT scan (sagittal). CT scan taken for radiation therapy planning. Line 2 indicates the level of the edge of the contoured prostatic apex. Line 1 indicates the level 5 mm cranial to Line 2, which is the level of the edge of the prostatic apex. Cases in which the hydrogel spacer could not be identified at the level of Line 1 on the MRI scan taken on the same day as the CT scan for treatment planning were defined as those with the hydrogel spacer not in place at the apex.

The patients in the Spacer group were further divided into those with the hydrogel spacer not in place at the prostatic apex (Non-Apex group) and those with the spacer in place at the apex (Apex group; Figure 2). In this study, cases in which no hydrogel spacer was observed on the slice at the level 5 mm cranial to the prostatic apex in the CTV, on the MRI scan taken on the same day as the first CT scan for treatment planning (Figure 3), were defined as the Non-Apex group. The rectal dose data (D2cc, D1cc, V70Gy, V60Gy, and V40Gy) for the Non-Spacer group and Non-Apex group were extracted from the treatment planning system and statistically analyzed using the Wilcoxon rank sum test.

## Results

A total of 81 patients underwent radical radiation therapy with IMRT for prostate cancer at Shimane University Hospital between March 2020 and June 2022. Of these, 45 patients were included in the Non-Spacer group and 36 patients in the Spacer group. The baseline characteristics of the patients in the Non-Spacer group and the Spacer group are shown in Table 1. Risk classification was based on the National Comprehensive Cancer Network guidelines 17. In the Non-Spacer group, 1 patient with lung metastasis at diagnosis and classified into the “metastasis” risk category was included in the “high-risk or more” category. In this patient, the lung metastasis resolved with hormonal therapy, with no other lesions present. Therefore, this patient received IMRT for the primary prostate cancer lesion, as with the other patients. The T stage distribution significantly differed between the two groups (P = 0.01), which may be attributable to the inclusion of factors related to T stage—such as extracapsular extension toward the dorsal side and seminal vesicle invasion—in the eligibility criteria for spacer implantation. No significant differences were observed between the two groups in other variables. Of the 36 patients in the Spacer group, 16 (44.4%) were classified into the Non-Apex group and 20 (55.6%) into the Apex group. The baseline patient characteristics of the Non-Apex group and Apex group are shown in Table 2. There were no significant differences in patient characteristics between the Apex and Non-Apex groups.

To address the first question, we analyzed whether the rectal dose was reduced by hydrogel-spacer implantation. The mean (± standard deviation) values of rectal dose parameters D2cc, D1cc, V70Gy, V60Gy, and V40Gy were 72.14 ± 3.56 Gy, 75.45 ± 1.68 Gy, 10.73 ± 2.80%, 17.00 ± 3.24%, and 31.16 ± 5.39%, respectively, in the Non-Spacer group and 60.07 ± 8.81 Gy, 66.10 ± 8.90 Gy, 3.41 ± 2.85%, 8.74 ± 4.72%, and 26.31 ± 5.69%, respectively, in the Spacer group. All rectal dose parameters were significantly lower in the Spacer group than in the Non-Spacer group (Table 3). We also analyzed the reduction in rectal dose data with and without hydrogel-spacer implantation in the same patients. Patients whose prostate volume change between the simulation plan and the treatment plan was within 20% were selected, resulting in 20 cases out of the total 36 in the Spacer group. The mean (± standard deviation) values of rectal dose parameters D2cc, D1cc, V78Gy, V70Gy, V60Gy, and V40Gy were 73.48 ± 3.77 Gy, 75.41 ± 2.87 Gy, 0.74 ± 1.15%, 10.84 ± 4.17%, 17.92 ± 3.78%, and 32.77 ± 6.37%, respectively, in the pre-spacer Group A, and 60.43 ± 9.80 Gy, 65.71 ± 9.66 Gy, 0.08 ± 0.16%, 3.45 ± 3.12%, 8.92 ± 5.09%, and 26.67 ± 6.24%, respectively, in the post-spacer Group B. All rectal dose parameters were significantly lower in Group B than in Group A.

For the second question, we analyzed whether the rectal dose could be reduced even if the hydrogel spacer was unevenly distributed in the craniocaudal direction. The rectal dose parameters D2cc, D1cc, V70Gy, V60Gy, and V40Gy for the Non-Apex group were 60.84 ± 7.66 Gy, 67.58 ± 8.17 Gy, 3.40 ± 2.53%, 8.62 ± 3.43%, and 26.67 ± 3.95%, respectively. All parameters were significantly lower in the Non-Apex group than in the Non-Spacer group (Table 4).

## Discussion

This study aimed to answer two questions. To address the first question, we compared the reduction in rectal dose with and without hydrogel-spacer implantation. The purpose of hydrogel-spacer implantation is to reduce AEs in the rectum, such as rectal bleeding. In a phase 3 study conducted by Hamstra et al. comparing IMRT with and without hydrogel spacers, the rectal V70Gy value was reduced by 25% or more in 97.3% of patients with an implanted hydrogel spacer 14. They also reported better outcomes in terms of rectal AEs in the hydrogel spacer group. Groups from other centers have also reported the effectiveness of hydrogel-spacer implantation 18,19. The present study, along with other reports, demonstrated that the rectal dose could be reduced by hydrogel-spacer implantation. A review of pelvic radiotherapy reported that the rectal volume receiving a dose of 60 Gy or more was correlated with the incidence of Grade ≥2 rectal AEs (according to the Radiation Therapy Oncology Group late radiation morbidity scoring scheme); that is, the higher the dose, the higher the incidence of AEs 10. The finding that the parameter values at high dose range, such as V60Gy and V70Gy, were significantly reduced in the Spacer group may be a good indication that hydrogel-spacer implantation can reduce rectal AEs. However, further follow-up is needed to ascertain whether the Spacer group will also have reduced late rectal toxicities compared with the Non-Spacer group in the setting of our hospital.

For the second question, we examined whether the rectal dose could be reduced in cases where the hydrogel spacer was unevenly distributed and not fully in place at the level of prostatic apex, compared with cases with no hydrogel-spacer implantation. In all cases, we implanted hydrogel spacers in accordance with the “Guidelines for Proper Use of SpaceOAR System in Radiation Therapy for Prostate Cancer” 16. When implanting a hydrogel spacer, the height of the needle tip was set at the center of the prostate in accordance with the proper use guidelines. However, despite the same implantation procedure being used, the distribution of the hydrogel spacers differed from case to case.

The effectiveness of hydrogel-spacer implantation in prostate cancer radiotherapy is generally accepted. However, the report by Hamstra et al. 14 did not examine the distribution of hydrogel spacer. Only a few reports have described the distribution of the hydrogel spacer and rectal AEs. A report by Fischer-Valuck et al. 20 discussed the distribution of hydrogel spacers, with a focus on their symmetry. Some degree of asymmetry was observed in 50.9% of cases, and a large deviation of more than 2 cm was observed in 1.3% of cases. However, except for the 1.3% of cases with large deviation, there was a significant reduction in rectal dose regardless of the asymmetric distribution of the hydrogel spacer. They therefore concluded that the asymmetric distribution of the hydrogel spacer is not an uncommon situation, and that, with the exception of cases with large deviation of the spacer, the implantation of the hydrogel spacer, even with asymmetric distribution, contributes to a reduction in the rectal dose. In the present study, we focused on the uneven distribution of the spacer in the craniocaudal direction, rather than in the left-right direction. We evaluated the rectal dose in those patients who underwent hydrogel-spacer implantation, but in whom the spacer was not in place at the prostatic apex. Of the 36 cases in which we implanted hydrogel spacers, in 16 cases (44%) the spacer was unevenly distributed in the craniocaudal direction, with the spacer not in place at the apex. The thickness of the hydrogel spacer tends to be thinner at the apex level than at the base or midgland level of the prostate 21, which is consistent with our cases. Fukumitsu et al. investigated the distribution and effectiveness of hydrogel spacers in proton beam therapy for prostate cancer 15. They found that the thickness of the hydrogel spacer was greatest in the center of the prostate and tended to gradually become thinner in the craniocaudal direction. In the discussion, they attributed the uneven distribution of the hydrogel spacer to the anatomical structure of the prostate and implantation method.

Regarding the positional relationship between the prostate and rectum after hydrogel-spacer implantation, studies have shown that the post-implantation distance between the prostate and rectum is smaller at the apex than at the center or base of the prostate 13,18. Even if the hydrogel spacer did not reach the apex, where the prostate and rectum are in close proximity, its implantation was still effective in reducing the rectal dose. Anatomically, the prostate is smaller in volume at the apex and larger at the center and base. The dose constraints for IMRT are usually optimized by volume, both for the target and for organs at risk. For this reason, the apex, which has a small volume, may have been relatively less affected throughout the treatment plan.

On the other hand, studies using different treatment modalities have reported that uniformly extending the spacer to the prostatic apex is effective in reducing the rectal dose 15,22. Fukumitsu et al. performed proton beam therapy, while Kobayashi et al. conducted stereotactic body radiotherapy (SBRT) for prostate cancer using the CyberKnife system. Our study was based on IMRT, and thus the treatment techniques differed. Proton beam therapy, as used by Fukumitsu et al., has physical properties different from those of photon-based radiotherapy. Proton beams exhibit a dose distribution known as the Bragg peak, which enables treatment with fewer irradiation fields and results in a dose distribution pattern substantially different from that of IMRT 23. Some studies comparing proton beam therapy and IMRT for prostate cancer have reported that proton beam therapy is superior in reducing the rectal dose 24. The SBRT used by Kobayashi et al. is a form of external beam irradiation with a small number of high-dose fractions, and has been shown to achieve oncological outcomes comparable to those of IMRT in prostate cancer treatment 25. The CyberKnife system, which employs a robotic arm-based linear accelerator, enables non-coplanar irradiation from multiple directions. Compared with IMRT, SBRT using the CyberKnife system has been noted to provide steeper dose gradients and superior reduction of rectal dose, particularly in the low-dose range 26. Both proton beam therapy and the CyberKnife system offer more favorable dose distributions than IMRT, and these differences in dose characteristics may partially explain the discrepancies between our findings and those of previous studies. Although proton beam therapy and the CyberKnife system represent excellent therapeutic options for prostate cancer, the number of centers able to provide these treatments remains limited compared with those providing IMRT. Therefore, the present study, which focused on IMRT that can be widely performed at many centers, provides clinically valuable information.

A Japanese survey found that 58.6% of centers did not perform hydrogel-spacer implantation 27. Implanting a hydrogel spacer was considered easy by 99% of clinicians, and has a very high success rate 13. Hydrogel-spacer implantation was reported to be effective even in regions where the procedure was not performed frequently 28. Our study showed that the hydrogel spacer did not reach the apex in about half of the cases, and Fischer-Valuck et al. 19 also reported left-right asymmetric distribution of the spacer in about half of cases. Despite the uneven distribution, hydrogel-spacer implantation was effective in reducing the rectal dose in both our study and the one reported by Fischer-Valuck et al. This suggests that hydrogel-spacer implantation is effective in reducing the rectal dose, even if the spacer is unevenly distributed in the left-right or craniocaudal direction. This may encourage centers that have not yet introduced hydrogel-spacer implantation due to a lack of technical proficiency to do so.

There are several limitations to this study. First, the follow-up period was too short for us to examine the effect of hydrogel-spacer implantation on late AEs. Based on the relationship between rectal dose and AEs, it is expected that a decrease in rectal dose will reduce AEs 9,10, but a longer-term follow-up is needed to confirm whether this is actually the case. Second, it was difficult to rigorously compare rectal dose reduction within the same patients. In some prospective studies 13,20, treatment-planning CT scans were obtained before spacer implantation, allowing direct pre- and post-implantation comparisons within the same patients. In contrast, our study was retrospective, and no treatment-planning CT scans immediately prior to spacer implantation were available. Therefore, diagnostic CT scans obtained at the time of prostate cancer diagnosis or other occasions were used to generate simulation plans for comparison. However, substantial differences in conditions, such as prostate size changes due to prior hormonal therapy, made accurate evaluation difficult. Although we attempted to reduce these differences by selecting cases with minimal size changes, other factors beyond the presence or absence of a spacer may still have influenced rectal dose. If treatment-planning CT scans immediately prior to spacer implantation had been available, the rectal dose-reducing effect could have been assessed more precisely. In future studies, treatment-planning CT scans should be acquired prior to spacer implantation to allow evaluation of rectal dose reduction within the same patients. The third limitation relates to the definition of the uneven distribution of the hydrogel spacer at the apex. There are various factors that describe the distribution of the hydrogel spacer, such as the degree of uneven distribution of the spacer in the craniocaudal direction and its thickness at the center of the prostate. If the definition of cases with the spacer not in place at the apex and the measurement method differ, then the analysis results will likely also differ.

Despite these limitations, the finding that hydrogel-spacer implantation was effective in reducing the rectal dose, regardless of the uneven distribution of the spacer, may encourage more centers to introduce hydrogel spacers, making this a valuable contribution to the literature.

## Conclusions

Hydrogel-spacer implantation was effective in reducing the rectal dose in IMRT for prostate cancer. Hydrogel-spacer implantation is often followed by uneven distribution of the spacer; nearly half of the cases had the hydrogel spacer not in place at the prostatic apex. Despite the uneven distribution of the hydrogel spacer, its implantation was still effective in reducing the rectal dose.

## Figures and Tables

**Table 1:** Characteristics of Non-Spacer group and Spacer group.

	Non-Spacer group (N=45)	Spacer group (N=36)	P-value
Age, median (range)	76 (62-86)	75 (60-84)	NS
Prostate Volume(mL), median(range)	27.2 (8.1-56.3)	28.3 (17.8-73.0)	NS
PSA(ng/mL), median(range)	10.4 (3.6-68.4)	10.1 (4.3-128.0)	NS
cT stage			0.01
cT1	2 (4.4%)	3 (8.3%)	
cT2	21 (46.7%)	27 (75.0%)	
cT3	20 (44.4%)	6 (16.7%)	
cT4	2 (4.4%)	0 (0.0%)	
Gleason score			NS
Grade Group 1	3 (6.7%)	1 (2.8%)	
Grade Group 2	7 (15.6%)	10 (27.8%)	
Grade Group 3	13 (28.9%)	10 (27.8%)	
Grade Group 4	15 (33.3%)	13 (36.1%)	
Grade Group 5	7 (15.6%)	2 (5.6%)	
Hormone therapy			
Yes/No	42 (93.3%) / 3 (6.6%)	31 (86.1%) /5 (13.9%)	NS
Risk group*			NS
Low risk	1 (2.2%)	1 (2.8%)	
Intermediate risk	16 (35.6%)	16 (44.4%)	
High risk or more	28 (62.2%)	19 (52.8%)	

NS, not significant (P > 0.05). *National Comprehensive Cancer Network guidelines Version 1.2025 December.4, 2024

**Table 2:** Characteristics of Non-Apex group and Apex group.

	Non-Apex group (N=16)	Apex group (N=20)	P-value
Age, median (range)	74 (60-84)	75.5 (60-84)	NS
Prostate Volume(mL), median(range)	34.4 (18.3-59.2)	25.7(17.8-73)	NS
PSA(ng/mL), median(range)	7.51(4.3-24)	10.1(4.3-128)	NS
cT stage			NS
cT1	1 (6.3%)	2 (10.0%)	
cT2	14 (87.5%)	13 (65.0%)	
cT3	1 (6.3%)	5 (25.0%)	
cT4	0	0	
Gleason score			NS
Grade Group 1	0 (0.0%)	1 (5.0%)	
Grade Group 2	4 (25.0%)	6 (30.0%)	
Grade Group 3	4 (25.0%)	6 (30.0%)	
Grade Group 4	7 (43.8%)	6 (30.0%)	
Grade Group 5	1 (6.3%)	1 (5.0%)	
Hormone therapy			
Yes/No	13 (81.3%) / 3 (18.8%)	18 (90.0%) / 2 (10.0%)	NS
Risk group*			
Low risk	0 (0.0%)	1 (5.0%)	NS
Intermediate risk	8 (50.0%)	8 (40.0%)	
High risk or more	8 (50.0%)	11 (55.0%)	

NS, not significant (P > 0.05). *National Comprehensive Cancer Network guidelines Version 1.2025 December.4, 2024

**Table 3:** Rectal dose parameters for patients with (Spacer group) and without (Non-Spacer group) hydrogel-spacer implantation. All parameters were significantly reduced in the Spacer group. (all p <0.05).

Rectal dose (mean ± Std Dev)	Non-Spacer group (N=45)	Spacer group (N=36)	P-value
D2cc (Gy)	72.1 ± 3.6	60.1 ± 8.8	<.0001
D1cc (Gy)	75.4 ± 1.7	66.1 ± 8.9	<.0001
V78Gy (%)	0.4 ± 0.4	0.1 ± 0.1	<.0001
V70Gy (%)	10.7 ± 2.8	3.4 ± 2.9	<.0001
V60Gy (%)	17.0 ± 3.2	8.7 ± 4.7	<.0001
V40Gy (%)	31.2 ± 5.4	26.3 ± 5.7	<.0001

Std Dev = standard deviation

**Table 4:** Rectal dose parameters for patients who did not undergo hydrogel-spacer implantation (Non-Spacer group) and those who did, but in whom the spacer was not in place at the prostatic apex (Non-Apex group). All parameters were significantly reduced in the Non-Apex group. (all p <0.05).

Rectal dose (mean ± Std Dev)	Non-Spacer group (N=45)	Non-Apex group (N=16)	P-value
D2cc (Gy)	72.1 ± 3.6	60.8 ± 7.7	<.0001
D1cc (Gy)	75.4 ± 1.7	67.6 ± 8.2	<.0001
V78Gy (%)	0.4 ± 0.4	0.1 ± 0.1	<.0001
V70Gy (%)	10.7 ± 2.8	3.4 ± 2.5	<.0001
V60Gy (%)	17.0 ± 3.2	8.6 ± 3.4	<.0001
V40Gy (%)	31.2 ± 5.4	26.7 ± 3.9	0.0038

Std Dev = standard deviation

## Abbreviations

IMRT: Intensity-modulated radiation therapy
3D-CRT: three-dimensional conformal radiation therapy
AEs: adverse events
CT: computed tomography
MRI: magnetic resonance imaging
CTV: clinical target volume
RTPS: radiotherapy treatment planning system
SBRT: stereotactic body radiotherapy
NS: not significant
Std Dev: standard deviation
